# Neuroendocrine tumor arising in Meckel’s diverticulum presenting with bowel obstruction

**DOI:** 10.1093/jscr/rjag025

**Published:** 2026-01-30

**Authors:** Jillian C Dawley, James H McClenathan, Belinda L Sun

**Affiliations:** Department of Pathology and Laboratory Medicine, Banner - University Medical Center, University of Arizona, 1625 N Campbell Ave, Tucson, AZ 85719, United States; Department of Surgery, Banner - University Medical Center, University of Arizona, 1625 N Campbell Ave, Tucson, AZ 85719, United States; Department of Pathology and Laboratory Medicine, Banner - University Medical Center, University of Arizona, 1625 N Campbell Ave, Tucson, AZ 85719, United States

**Keywords:** Meckel’s diverticulum, small bowel obstruction, neuroendocrine neoplasm, neuroendocrine tumor

## Abstract

Meckel’s diverticulum (MD) is a common developmental abnormality of the gastrointestinal tract that, in adults, is usually asymptomatic but can present with complications including malignancy. Although malignancy in MD is rare overall, MD is recognized a high-risk site for tumorigenesis compared to the surrounding ileum. Neuroendocrine neoplasms are the most common malignancy arising in MD and are often associated with nodal metastases, even if the primary tumor is small. We report the case of a 66-year-old male who underwent exploratory laparotomy for small bowel obstruction and was intraoperatively found to have MD causing an internal hernia. Pathologic examination of the diverticulectomy specimen revealed a well-differentiated neuroendocrine tumor, grade 1, pathologic stage pT2. The case highlights the role of resection for incidentally found and symptomatic MD due to the elevated risk of malignancy at this site.

## Introduction

Meckel’s diverticulum (MD) is a true diverticulum of the small intestine, usually located in the ileum, resulting from incomplete closure of the vitelline duct. MD represents the most common developmental abnormality of the gastrointestinal tract and in the adult is usually asymptomatic, but can present with complications of bleeding, obstruction, diverticulitis, perforation, and, rarely, sequelae of tumors [1]. Though malignancy in MD is overall rare, MD is a high-risk region for tumorigenesis, with a reported adjusted risk of cancer 70 times time higher than any other ileal site [2]. Neuroendocrine neoplasms (NENs) are the most common neoplasms arising in MD, and reports suggest that MD NENs, even if small and/or well-differentiated, behave aggressively [3].

Herein, we present the case of a 66-year-old male who presented with small bowel obstruction with intraoperative findings of MD causing an internal hernia. Histopathologic examination of the resected MD revealed the presence of a well-differentiated neuroendocrine tumor (NET).

## Case report

### Clinical history

A 66-year-old male with no significant past medical history presented with intermittent episodes of bright red and coffee-ground emesis, dark stools, and abdominal pain. He had no prior history of abdominal surgeries or gastrointestinal bleeding. Physical examination of the abdomen showed mild distension and tenderness to palpation without peritoneal signs. Laboratory studies were notable for neutrophilic leukocytosis (white blood cell count [WBC] 24.7 K/μL), normocytic anemia (Hgb 11.7 g/dL and mean corpuscular volume [MCV] 86 fL), and thrombocytosis (1,008 K/μL). Computed tomography (CT) of the abdomen/pelvis showed distal small bowel obstruction and mild ascites, with no masses or lesions noted.

The patient’s small bowel obstruction was initially managed conservatively with nasogastric tube decompression, however after lack of resolution, he underwent exploratory laparotomy. Intraoperative findings included MD with an omphalomesenteric band connecting to the umbilicus causing an internal hernia. Meckel’s diverticulectomy with division and ligation of the omphalomesenteric band were performed.

The patient had an uncomplicated post-operative course following his abdominal surgery. He continued to have intermittently worsening anemia suggestive of ongoing gastrointestinal bleeding that was managed medically and ultimately stabilized. Upper GI endoscopy performed two days following the surgery showed non-bleeding esophagitis and one non-bleeding, superficial ulcer in the gastric body. The patient was discharged in a stable condition.

### Pathologic findings

Gross examination of the diverticulectomy specimen showed a small bowel diverticulum, 4.1 cm in length and up to 2.2 cm in diameter, with minimal attached mesenteric fat. Sectioning revealed a 1.1 × 0.9 × 0.7 cm tan-white, sessile mass, 0.2 cm from the resection margin. The mass grossly appeared to abut the muscularis propria.

**Figure 1:**
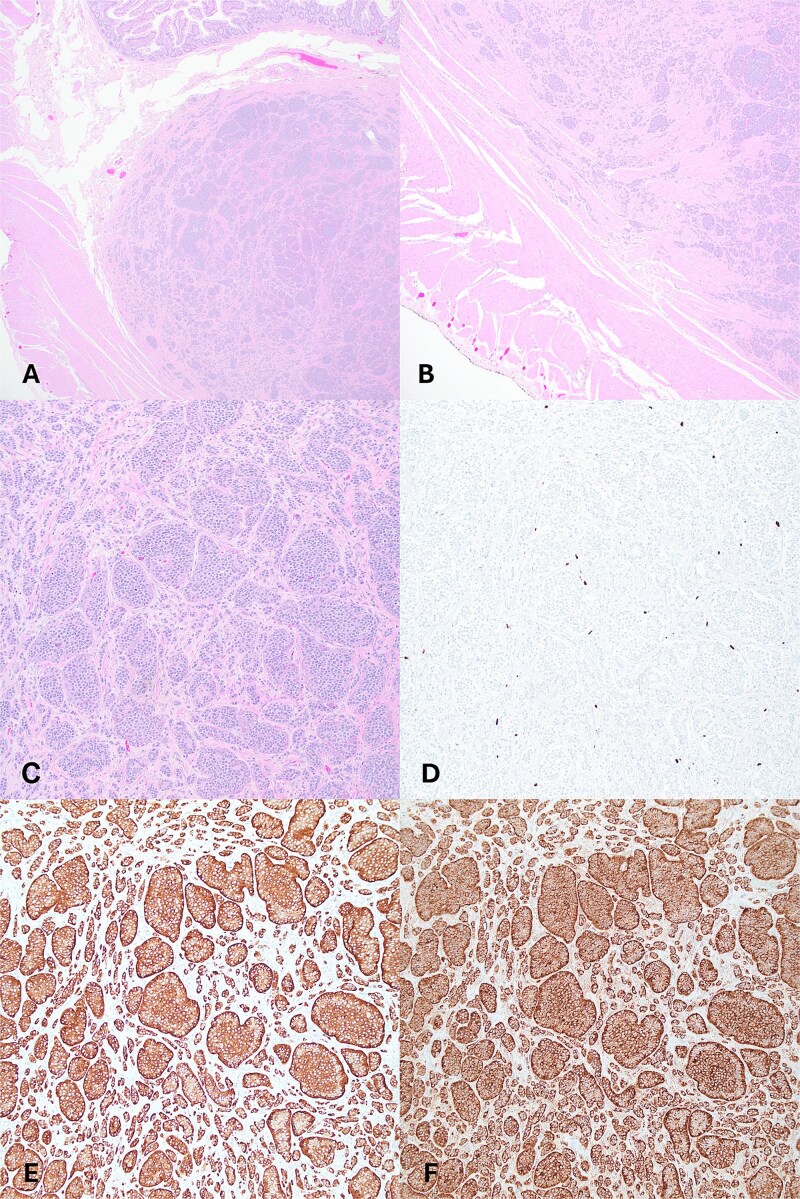
(A) Tumor present in submucosa of Meckel’s diverticulum, H&E 20x. (B) Tumor with microscopic invasion of muscularis propria, H&E, 40x. (C–F) Tumor shows well-differentiated neuroendocrine cells with organoid growth pattern (C), low Ki67 proliferation pattern (D), immunohistochemistry positivity for chromogranin (E), synaptophysin (F); (A–F) 100x.

Microscopic examination showed a well-differentiated NET invading the submucosa with focal invasion of the muscularis propria (Fig. 1A and B). The tumor comprised uniform cells with round to oval nuclei and finely granular chromatin, arranged predominantly in nests and acini (Fig. 1C). No lymphovascular invasion was identified. The background diverticulum showed small bowel and gastric-type mucosa. No lymph nodes were present. Immunohistochemistry demonstrated the tumor to be strongly positive for chromogranin and synaptophysin. The mitotic count was 0.2/2 mm^2^ and the Ki67 index was 1%. Taken together, the patient was diagnosed with a well-differentiated NET, grade 1, pathologic stage pT2.

## Discussion

Malignancy is a rare complication of MD, with reported incidence of 0.5%–5.1% [1, 2]. Even so, MD is recognized as a high-risk region for tumorigenesis when compared with the risk in non-Meckel ileal tissue [2]. NETs account for approximately two thirds of malignancies arising in MD [1].

There is not clear consensus regarding surgical management of asymptomatic MD in adults [1, 4]. Thirunavukarasu et al. performed a large review of the comparing malignancy in MD to other ileal malignancy, including 121 NETs, and recommended routine resection due to elevated risk of cancer [2]. A more recent literature analysis suggested an approach based on risk factors for complications including male gender, age less than 40 years, size greater than 2 cm, and macroscopically evident heterotopic mucosa [5].

Symptomatic MD are treated with resection [1]. Our case of MD NET was discovered due small bowel obstruction, and the association of the patient’s upper gastrointestinal bleed, MD, and NET is unclear. There is a recent case report of a NET arising in MD presenting as an upper GI bleed with tumor thought to be the source of bleeding, rather than ectopic gastric mucosa [6].

Multiple authors have stressed the importance of palpating for masses at time of MD resection to guide appropriate surgical management [7, 8]. Highlighting this point, Bacalbasa et al. reported a case of MD found incidentally during inguinal hernia repair that, due to the presence of a palpable ectopic tissue, was widely resected with adjacent ileal segment and was found to have a 0.6 cm, well-differentiated NET invading the submucosa [9]. Despite the typical presentation of NETs in males with a median age of 50, NETs have been reported in children, including a recent report of a 4 mm NET in the MD of a 5-year-old female who presented with recurrent intussusception [10].

Several studies have shown that NETs arising in MD have a high risk of metastasis regardless of size. In a case series of 7 MD NETs, 6 patients had regional lymph node involvement, of which 3 had primary tumors <2 cm [7]. Dogeas et al. performed a population-based study of 280 MD NETs including 87 surgically resected tumors with known status of regional lymph node metastasis and found that sub-centimeter MD NET carried a 26.5% risk of nodal metastasis, compared to 53.6% and 73.7% for tumors 1–2 cm and > 2 cm in size, respectively [3]. The authors also demonstrated a significant rate of nodal metastasis (41%) among well-differentiated tumors [3]. These findings support regional lymphadenectomy for NETs arising in MD.

In the present case, the tumor was well-differentiated, low grade, and small in size, however exhibited invasion of muscularis propria. Unfortunately, our patient was lost to follow up without having undergone regional lymphadenectomy. The case emphasizes the role of resection of MD due to elevated risk of malignancy such as NET and the aggressive behaviour of NETs arising in this site.

## References

[1] Van Malderen K, Vijayvargiya P, Camilleri M et al. Malignancy and Meckel's diverticulum: a systematic literature review and 14-year experience at a tertiary referral center. United European Gastroenterol J 2018;6:739–47. 10.1177/2050640617752771 PMC6068795 30083336

[2] Thirunavukarasu P, Sathaiah M, Sukumar S et al. Meckel's diverticulum--a high-risk region for malignancy in the ileum. Insights from a population-based epidemiological study and implications in surgical management. Ann Surg 2011;253:223–30. 10.1097/SLA.0b013e3181ef488d 21135700 PMC4129548

[3] Dogeas E, Magallanes M, Porembka MR et al. Neuroendocrine tumors in Meckel's diverticulum: recommendation for lymphadenectomy regardless of tumor size based on the NCDB experience. J Gastrointest Surg 2019;23:679–85. 10.1007/s11605-018-04096-7 30706377

[4] Kato S, Saito T, Kurahashi S et al. Simultaneous resection of a neuroendocrine tumor in an incidental Meckel's diverticulum with transabdominal preperitoneal hernial repair: a case report. Surg Case Rep 2024;10:21. 38231465 10.1186/s40792-024-01821-0 PMC10794676

[5] Lequet J, Menahem B, Alves A et al. Meckel's diverticulum in the adult. J Visc Surg 2017;154:253–9. 10.1016/j.jviscsurg.2017.06.006 28698005

[6] Eberly KE, Martinez J, Sardana N. Unusual upper gastrointestinal bleeding due to a neuroendocrine tumor arising in Meckel diverticulum. ACG Case Rep J 2024;11:e01345. 10.14309/crj.0000000000001345 38638202 PMC11025707

[7] Lorenzen AW, O'Dorisio TM, Howe JR. Neuroendocrine tumors arising in Meckel's diverticula: frequency of advanced disease warrants aggressive management. J Gastrointest Surg 2013;17:1084–91. 10.1007/s11605-013-2191-8 23558715 PMC4438262

[8] Sugezawa K, Saito H, Kono Y et al. Neuroendocrine tumor arising from Meckel's diverticulum unexpectedly diagnosed after diverticulectomy and in which multiple lymph node metastases were found after reoperation: a case report. Yonago Acta Med 2018;60:251–4. 29434496 10.24563/yam.2017.12.007 PMC5803163

[9] Bacalbasa N, Costin R, Orban C et al. Incidental finding of a neuroendocrine tumor arising from Meckel diverticulum during hernia repair: a case report and literature review. Anticancer Res 2016;36:1861–4. 27069171

[10] Naji H, Allateef R, Ahmed A et al. Recurrent intussusception in a child revealing a neuroendocrine tumor within a Meckel's diverticulum: a case report. J Surg Case Rep 2025;2025:rjaf085. 10.1093/jscr/rjaf085 40040771 PMC11879116

